# Growth and production of bioactive compounds are boosted in young basil plants grown under hydroponic vitamin supplementation

**DOI:** 10.3389/fnut.2026.1840106

**Published:** 2026-07-15

**Authors:** Gabriela Rodrigues Sant’Ana, Alana Silva Rocha, Flávio Ferreira da Silva Binotti, Claudia Andrea Lima Cardoso, Sidney Mariano dos Santos, Eliana Duarte Cardoso Binotti, Carlos Eduardo da Silva Oliveira, Gilda Carrasco, Sebastião Ferreira de Lima, Edilson Costa, Eduardo Pradi Vendruscolo

**Affiliations:** 1 Postgraduate Program in Agronomy - Sustainability in Agriculture, State University of Mato Grosso do Sul – UEMS, Cassilândia, Mato Grosso do Sul, Brazil; 2 Center of Studies in Natural Resources, Postgraduate Program in Natural Resources, State University of Mato Grosso do Sul, Dourados, Mato Grosso do Sul, Brazil; 3 Departamento de Horticultura, Facultad de Ciencias Agrarias, Universidad de Talca, Talca, Chile; 4 Department of Agronomy, Federal University of Mato Grosso do Sul, Chapadão do Sul, Mato Grosso do Sul, Brazil

**Keywords:** antioxidant activity, gas exchange, hydroponic cultivation, *Ocimum basilicum*, plant biostimulants, secondary metabolites

## Abstract

**Background:**

Basil is a high-value aromatic and medicinal plant widely recognized for its rich composition of bioactive compounds with applications in food, pharmaceutical, and cosmetic industries. This study aimed to evaluate the physiological responses, growth, and production of bioactive compounds in baby leaf basil cultivated in a hydroponic system supplemented with B-complex vitamins.

**Methods:**

The experiment was conducted under a controlled environment using a randomized block design with four treatments: control, nicotinamide, thiamine, and pyridoxine. Plants were assessed 14 days after sowing for gas exchange parameters, biometric traits, biomass accumulation, leaf pigments, and bioactive compounds, including phenolics, flavonoids, tannins, antioxidant activity, and sun protection factor.

**Results:**

Vitamin supplementation reduced intercellular CO_2_ concentration and significantly increased CO_2_ assimilation rate, water use efficiency, and carboxylation efficiency. Nicotinamide notably enhanced plant growth, increasing leaf area (78%) and plant height (34.8%), as well as shoot fresh and dry mass (49.7 and 77.9%). Bioactive compound accumulation was also stimulated, with increases of up to 129.1% in phenolics and 159.2% in antioxidant activity, with nicotinamide being superior to the other treatments in all nutraceutical characteristics. Additionally, chlorophyll and carotenoid contents were elevated, especially under nicotinamide treatment, which also reduced leaf temperature. Pyridoxine showed intermediate effects, while thiamine presented modest improvements compared to the control.

**Conclusion:**

Hydroponic supplementation with B vitamins, particularly nicotinamide, enhances physiological performance, biomass production, and the accumulation of bioactive compounds in basil.

## Graphical abstract

**Figure:**
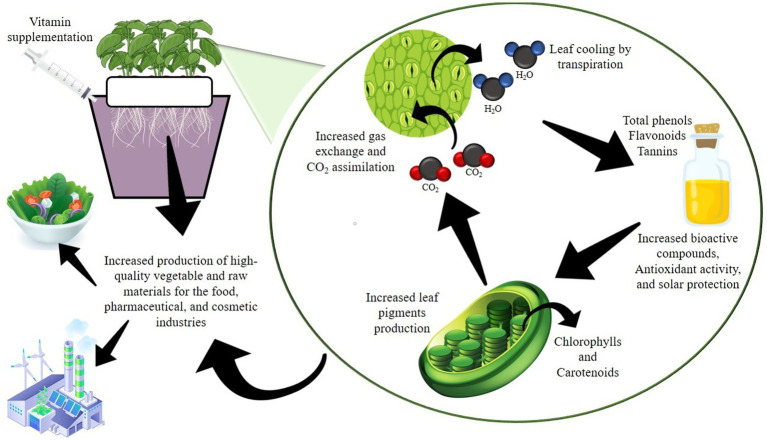

## 1 Introduction

Basil (*Ocimum basilicum* L.) is a species native to India, Africa, and South Asia, belonging to the Lamiaceae family and the Ocimum genus. It is a widely recognized plant among aromatic and medicinal plants (AMPs), characterized by its high added value and the diversity of varieties available on the market (1). This herb has a chemical composition rich in secondary metabolites, such as tannins, phenolic compounds, flavonoids, anthocyanins, carotenoids, steroids, and essential oils, which are of significant interest to the pharmaceutical, cosmetic, and food industries, given their applicability in the formulation of high-value-added products (2, 3).

In addition to its aromatic and medicinal potential, basil also stands out for its nutritional profile, being an excellent source of vitamins A and C, as well as essential minerals, including magnesium, calcium, iron, zinc, and potassium (4). These components reinforce the importance of this species in human nutrition, especially in the context of diets that seek nutritional diversity and functional properties. However, the amount of compounds in this crop can vary according to factors that influence plant development, such as climatic conditions, genotype, maturity, type of cultivation (5), or even the addition of elicitor and biostimulant compounds, including vitamins (6, 7).

The exogenous application of B vitamins, such as nicotinamide, thiamine, and pyridoxine, has been associated with increased levels of photosynthetic pigments and compounds with antioxidant activity, favoring the stability of physiological functions in both, stressful situations (8, 9), and in situations where plants are in suitable conditions, such as environments with partial control of climatic variables (10). These effects are mainly related to the protective function, with stimulation of secondary metabolism, activation of antioxidant enzymes, and accumulation of free proline, contributing to the integrity of the photosynthetic apparatus and the reduction of cellular damage (11). However, the activation of these protective mechanisms, when under adequate health conditions, results in the biostimulation of plants through the optimization of physiological processes, often becoming indistinguishable (12).

As a consequence of optimizing gas exchange capacity and the growth of vegetative and reproductive organs, increases in growth and productivity are observed, as reported for melon (13) and lettuce (28), both grown in hydroponic systems. Furthermore, an increase in the efficiency related to the use of atmospheric CO_2_ is observed, increasing its use as a substrate during the incorporation of dry matter (14).

Based on evidence of the positive effects of vitamins on plant development, the objective of this study was to evaluate the physiology, growth, and composition of basil grown as baby leaf in a hydroponic system, under the supply of B complex vitamins (nicotinamide, thiamine, and pyridoxine).

## 2 Materials and methods

### 2.1 Characterization of the experimental environment

The growing environment consisted of a climate-controlled greenhouse measuring 14.64 m × 6.40 m × 3.5 m (93.70 m^2^) plus an antechamber measuring 3.66 m × 3.20 m (11.71 m^2^), for a total area of 105.41 m^2^. The roof and sides of the greenhouse were covered with a double-layer, light-diffusing, 150-micron low-density polyethylene (LDPE) film, with a 1.2 m × 0.15 m Humil Cool (CELDEX®) pad/fan climate control system. A movable, aluminized ALUMINET® 35% shade screen was placed under the polyethylene film.

**Figure 1:**
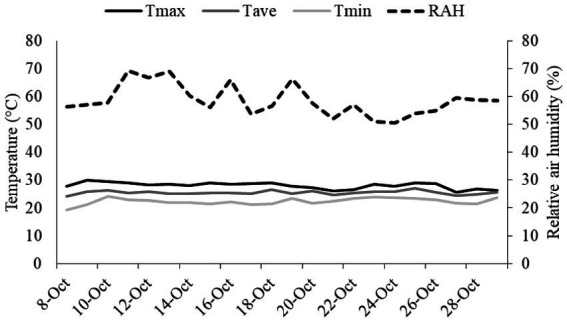
Maximum, average, and minimum temperature conditions and relative humidity during the experiment period.

Microclimatic data for maximum, average, and minimum temperatures, as well as relative air humidity for the experimental period, were obtained from a weather station installed in the center of the growing environment (Figure 1). The average maximum and minimum temperatures and relative air humidity during the experiment were 28 °C, 22.4 °C, and 58.6%, respectively.

### 2.2 Experimental design

The experimental design used was a randomized block design with four treatments and five replications. The treatments consisted of the application of bio-inputs based on: T1 – control; T2 – nicotinamide (50 mg L^−1^); T3 – thiamine (50 mg L^−1^), and T4 – pyridoxine (50 mg L^−1^). Each experimental plot consisted of a black polyethylene container containing a tray with 50 polystyrene cells (3.5 × 3.5 cm), filled with commercial substrate, containing three basil plants per cell. Each replicate consisted of five cells, each containing three plants. The definition of the vitamins and their concentrations was achieved through preliminary tests.

### 2.3 Implementation and execution of the experiment

Basil seeds of the “Basilicão” cultivar., from the company Isla Seeds, were sown in cells of trays containing commercial substrate Carolina Soil®. After sowing, the set was placed in an 11-liter container, characterizing a floating system, containing a 50% concentration nutrient solution [18% nitrogen (N), 8% phosphorus (P), 30% potassium (K), 15% calcium (Ca), 3% sulfur (S), 3% magnesium (Mg), 0.14% iron (Fe), 0.04% boron (B), 0.04% manganese (Mn), 0.03% copper (Cu), 0.019% molybdenum (Mo), 0.006% nickel (Ni), 0.002% cobalt (Co)] containing the treatments. For the application of vitamins in the solution, they were diluted directly in the nutrient solution, which was maintained with an electrical conductivity of 0.75 dS m^−1^ and a pH between 6.0 and 6.5, and changed at 7 and 14 days after sowing.

Evaluations were conducted 21 days after sowing. At this time, physiological assessments were performed on the plants using an infrared gas analyzer (IRGA, LCi, ADC BioScientific, Hertfordshire, UK) to determine variables related to gas exchange. During the measurement, the average leaf temperature was 25 °C, the CO_2_ concentration was 440 μmol mol^−1^, and the light intensity was 1,000 μmol m^−2^ s^−1^ of photosynthetic photochemical flux density. Readings were obtained for the characteristics of CO₂ concentration in the leaf mesophyll (Ci), transpiration rate (E), stomatal conductance (gS), and CO₂ assimilation rate (A). Water use efficiency (WUE) and carboxylation efficiency (EICi) were also calculated, which are derived from the ratio between A/E and A/Ci, respectively.

Next, the following biometric analyses were performed: number of leaves, stem diameter, shoot height, root length, shoot fresh weight, shoot dry weight, and leaf area. Shoot height and root length were measured with a graduated ruler, while stem diameter was determined with a digital caliper. Leaf area was obtained using the Easy Leaf Area software (15). Shoot fresh weight was determined using a precision digital scale, and subsequently, the plant material was dried in a forced-air chamber at 40 °C until a constant mass was obtained and weighed on a precision digital scale to obtain the dry mass.

The dried material was subjected to extraction with 70% ethanol using 10% plant material. The extraction took place at room temperature (20 ± 1 °C) for 48 h (29).

The phenolic content was determined using the Folin–Ciocalteu method (29). A reaction mixture was prepared with 1 mL of the sample, 0.5 mL of Folin–Ciocalteu reagent (1:10 v:v), and 1 mL of distilled water. After reacting for 1 min, 1.5 mL of 20% (w:v) sodium carbonate was added. The mixture was incubated at room temperature for 120 min, and the absorbance was measured at 760 nm using a UV/Vis spectrophotometer (UV-M51, BEL Photonics, Brazil). Quantification was based on a gallic acid calibration curve (10–1,000 μg mL^−1^; *y* = 0.0015x + 0.0008, *R*^2^ = 0.9875), and results were expressed as μg gallic acid equivalent per mL of extract (μg GAE mL^−1^). A blank was prepared by replacing the sample with the extraction solvent. The test was performed in triplicate.

The flavonoid content was determined using an assay with 2% (w/v) aluminum chloride (29). Equal volumes (1 mL) of aluminum chloride solution and the sample were mixed. After 15 min at room temperature, the absorbance was read at 430 nm using a UV/Vis spectrophotometer. Quantification was based on a rutin calibration curve (10^−50^ μg mL^−1^; y = 0.0105x + 0.0019, *R*^2^ = 0.9990), and the results were expressed as μg of rutin equivalent per mL of extract (μg ER mL^−1^). A blank was prepared by replacing the sample with the extraction solvent. The test was performed in triplicate.

The tannin content was determined using the Folin–Denis method (30). Equal volumes (0.5 mL) of Folin–Denis solution and the sample were mixed, and after 3 min, 0.5 mL of 8% (w/v) sodium carbonate was added to the reaction mixture. After 120 min at room temperature, the absorbance was read at 725 nm using a UV/Vis spectrophotometer. Quantification was based on a tannic acid calibration curve (0.5–80 μg mL^−1^; *y* = 0.009x + 0.03837, *R*^2^ = 0.99826), and the results were expressed as μg of tannic acid equivalent per mL of extract (μg EAT mL^−1^). A blank was prepared by replacing the sample with the extraction solvent. The test was performed in triplicate.

To evaluate the antioxidant potential of each 100 μL sample, 2,000 μL of a 0.1 mmol L^−1^ DPPH solution in methanol was added. The reaction was conducted for 30 min protected from light, followed by a UV/Vis spectrophotometer reading at a wavelength of 517 nm. The percentage of inhibition was calculated according to the equation: %I = 100 × (AbsC − AbsA)/AbsC, where %I represents the inhibition (%), AbsC is the absorbance of the control, and AbsA is the absorbance of the sample after the reaction. The control was performed by replacing the sample with the extraction solvent. The test was performed in triplicate.

The extracts obtained were analyzed using a UV/Vis spectrophotometer at wavelengths of 470, 649, and 665 nm. Chlorophyll a (Ca) was calculated using Equation 1, chlorophyll b (Cb) was calculated using Equation 2, and carotenoids (Cx + c) were calculated using Equation 3 (31).

$$\mathrm{Ca}=13.95\times A_{665}-6.88\times A_{649}$$ (1)

$$\mathrm{Cb}=24.96\times A_{649}-7.32\times A_{665}$$ (2)

$$\mathrm{Cx}+c=(1,000\times A_{470}-2.05\times \mathrm{Ca}-114.8\times \mathrm{Cb})/245$$ (3)

The samples were scanned in a UV/Vis spectrophotometer at wavelengths from 290 to 320 nm with 5 nm intervals. The sun protection factor (SPF) was calculated using Equation 4. FC represents the correction factor, EE*λ* × *Iλ* represents the erythemal efficiency spectrum per wavelength, and Abs*λ* represents the absorbance spectrum of the sample (32). The FC used was 10. The EE values were taken from the work of Sayre et al. (33).

$$\mathrm{SPF}=\mathrm{FC}\times \sum_{290}^{320}{\mathrm{EE}}_{\lambda }\times I_{\lambda }\times {\mathrm{Abs}}_{\lambda }$$ (4)

### 2.4 Statistical analysis

The data obtained were subjected to preliminary tests of normality and homoscedasticity, followed by analysis of variance (ANOVA), and compared using Tukey’s test at a 5% probability level. The data were also subjected to analysis to determine the network Pearson correlation (R software version v. 4.3.3 - qgraph package), through the correlation matrix. Multivariate analysis was performed using canonical variables (Candisc package), applied in the R software (16).

Multivariate visualization was performed using a radar chart (spider plot) with the R software (16) and the packages fmsb, dplyr, and scales. Data were initially grouped by treatment, and the means of the analyzed variables were calculated. Subsequently, the values were normalized to a percentage scale (0–100%) to allow comparison between variables with different units.

## 3 Results

**Figure 2:**
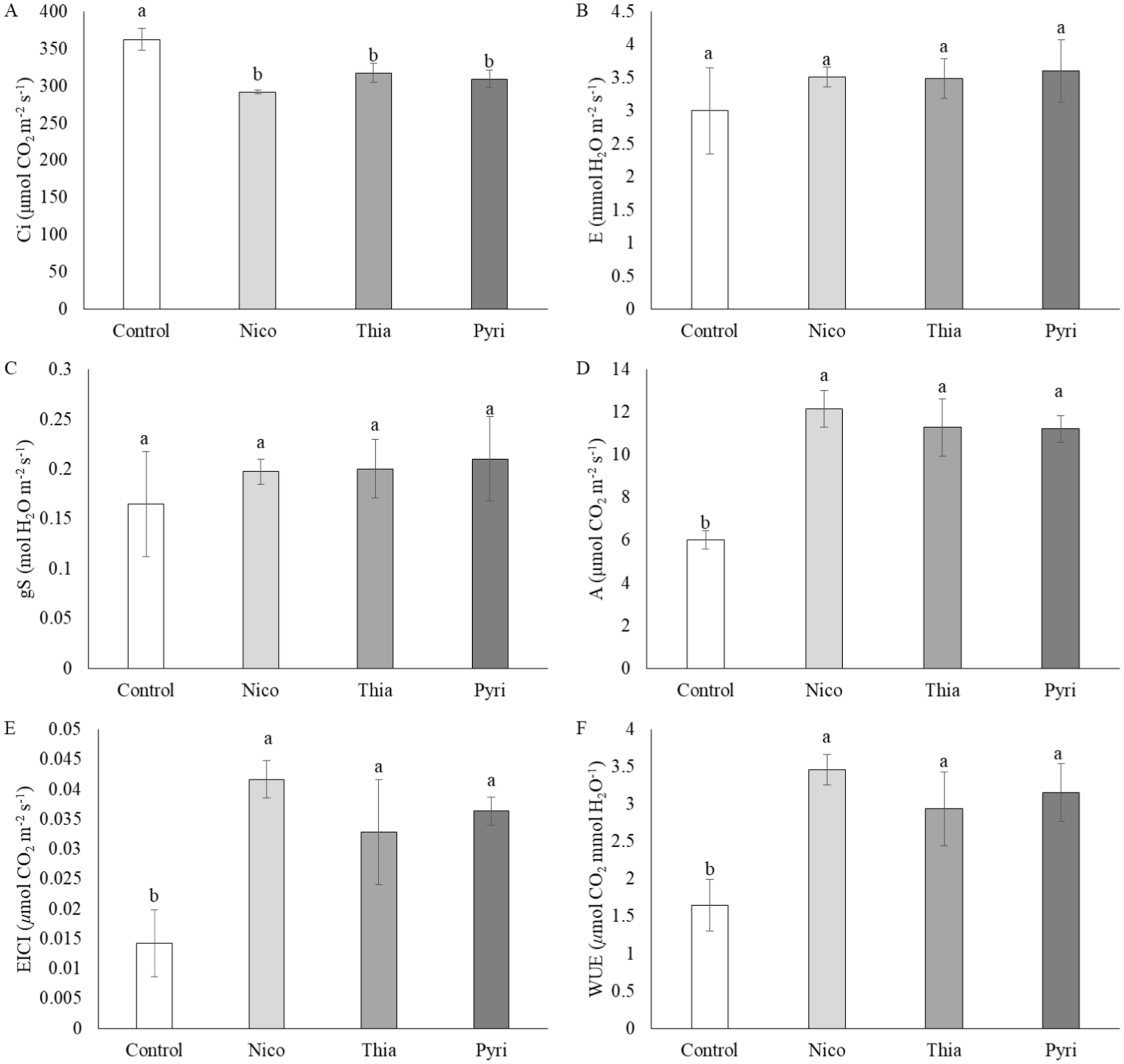
CO₂ concentration in the leaf mesophyll - Ci **(A)**, transpiration rate - E **(B)**, stomatal conductance - gS **(C)**, CO₂ assimilation rate - A **(D)**, carboxylation efficiency - EICi **(E)**, and water use efficiency - WUE **(F)** of babyleaf basil grown under different vitamin treatments. Different letters on the bars indicate a significant difference between the means by the Tukey test (*p* < 0.05). Bars represent the mean values (*n* = 4 ± SD).

It was found that the application of vitamins significantly reduced the concentration of CO_2_ in the leaf mesophyll (Figure 2A), while not affecting transpiration and stomatal conductance (Figures 2B,C). In addition, there was a marked increase in the CO_2_ assimilation rate (Figure 2D). In this sense, the average decrease in CO_2_ concentration in the leaf mesophyll was 15.6%, while the average increase in the assimilation rate obtained with the application of vitamins was 92.2%. For carboxylation efficiency and water use efficiency, the three vitamins resulted in superiority to the control treatment (Figures 2E,F), with average gains of 83.3 and 93.4%, respectively, for these variables.

**Figure 3:**
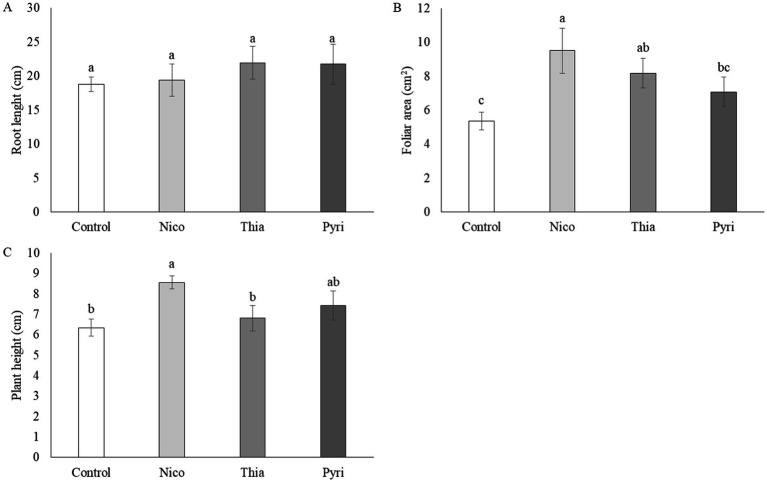
Root length **(A)**, foliar area **(B)**, and plant height **(C)** of babyleaf basil grown under different vitamin treatments. Different letters on the bars indicate a significant difference between the means by the Tukey test (*p* < 0.05). Bars represent the mean values (*n* = 4 ± SD).

No significant differences were observed for root length (Figure 3A). However, for leaf area and plant height, the treatment with nicotinamide application was found to be superior (Figures 3B,C). Compared to the control treatment, nicotinamide provided gains of 78% in leaf area and 34.8% in plant height.

**Figure 4:**
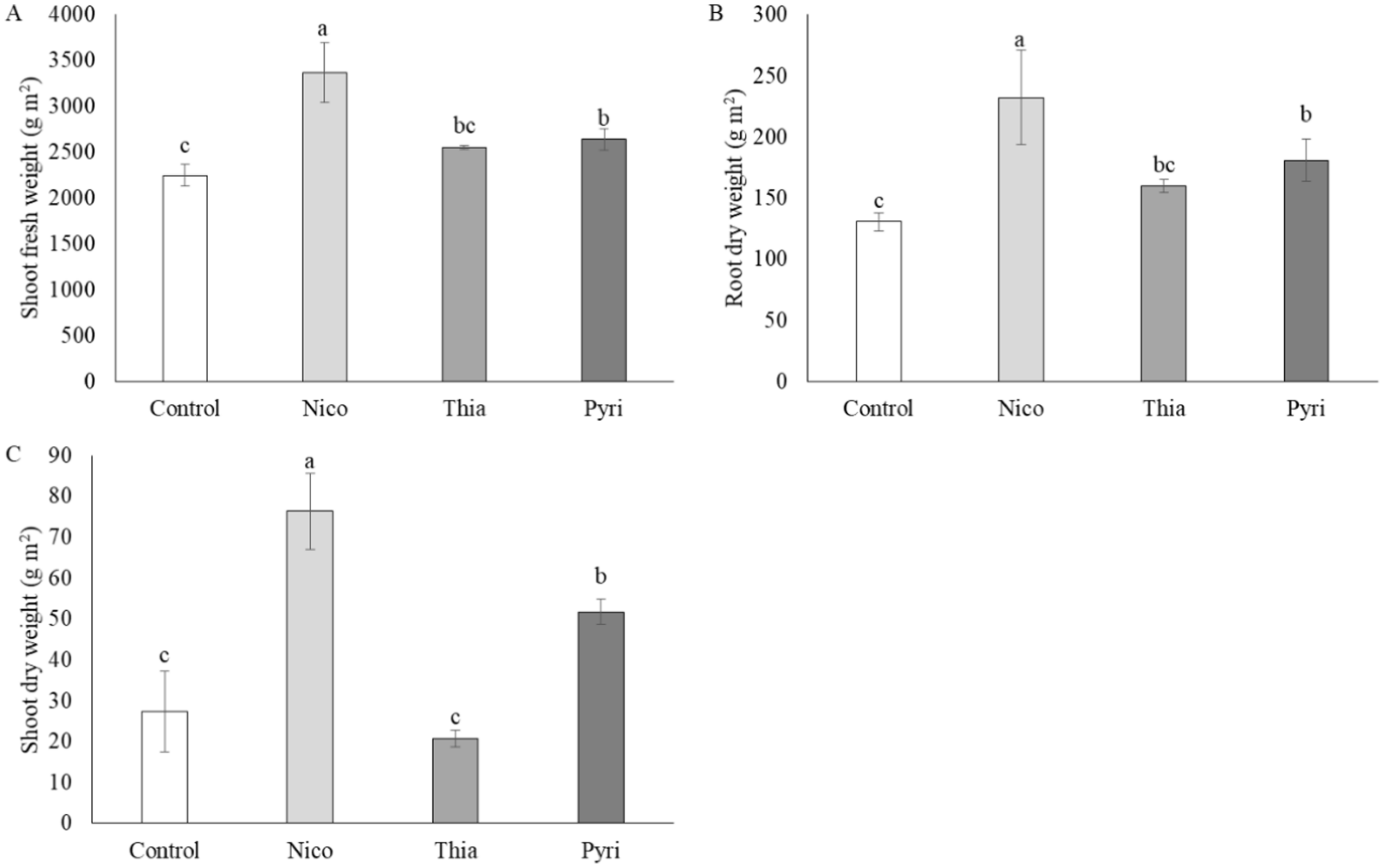
Shoot fresh weight **(A)**, root dry weight **(B)**, and shoot dry weight **(C)** of babyleaf basil grown under different vitamin treatments. Different letters on the bars indicate a significant difference between the means by the Tukey test (*p* < 0.05). Bars represent the mean values (*n* = 4 ± SD).

The use of nicotinamide in the nutrient solution also resulted in superior results over the other treatments for fresh shoot mass, root dry mass, and shoot dry mass (Figures 4A–C). For these variables, the superiority of the nicotinamide treatment was 49.7, 178.7, and 77.9%, respectively. In addition, the pyridoxine treatment also differed from the control treatment, following the nicotinamide treatment. Thus, the use of pyridoxine resulted in increases of 17.5, 88.7, and 38.5%, respectively.

**Figure 5:**
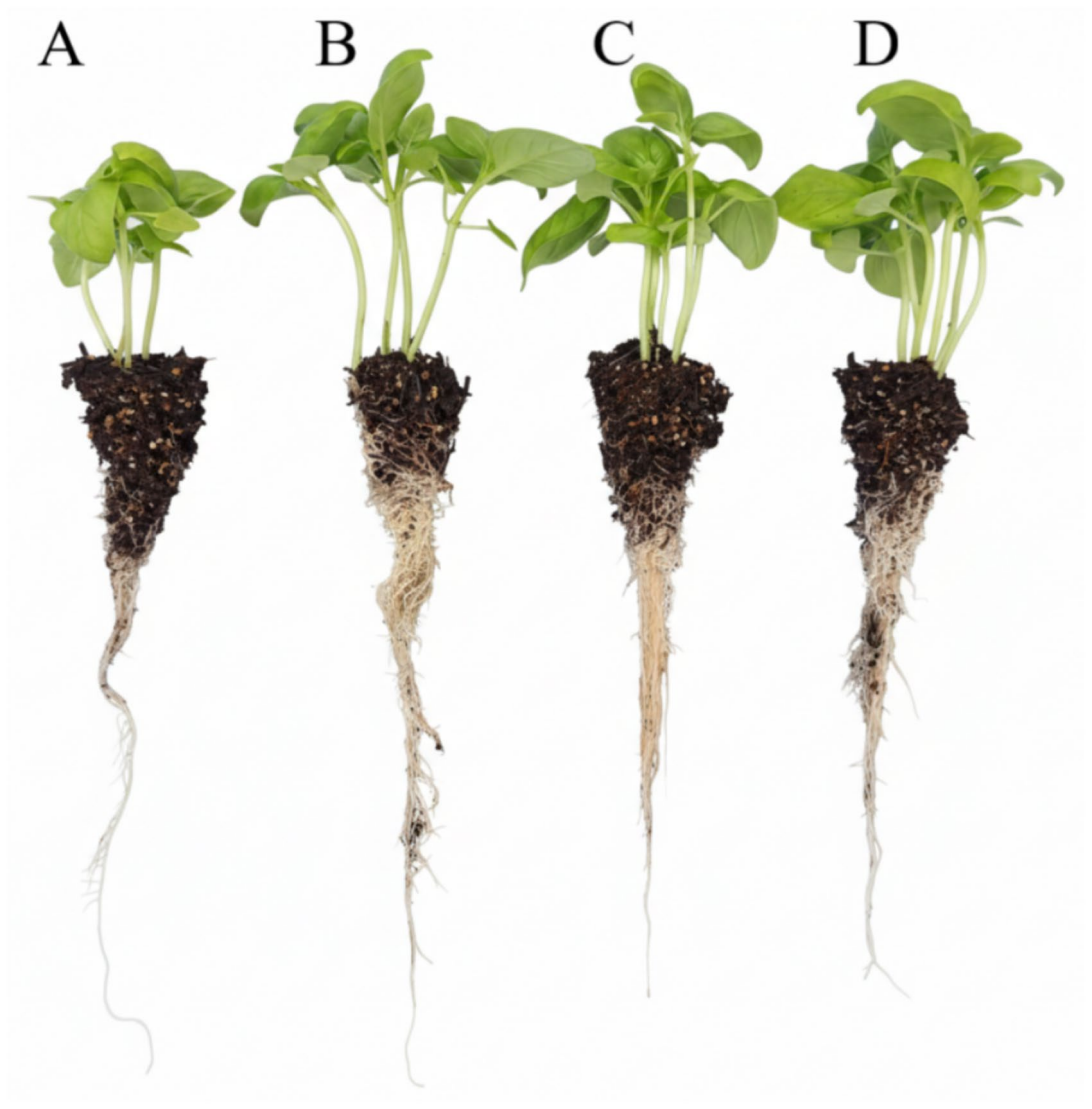
Comparative image of the babyleaf basil plants grown under different vitamin treatments, control **(A)**, nicotinamide **(B)**, thiamine **(C)**, and pyridoxine **(D)**.

The increased vigor in the development of the shoot and roots could be clearly seen, highlighting the development of the leaf blade and the volume of the roots of the plants grown in solution with the presence of vitamins (Figure 5).

**Figure 6:**
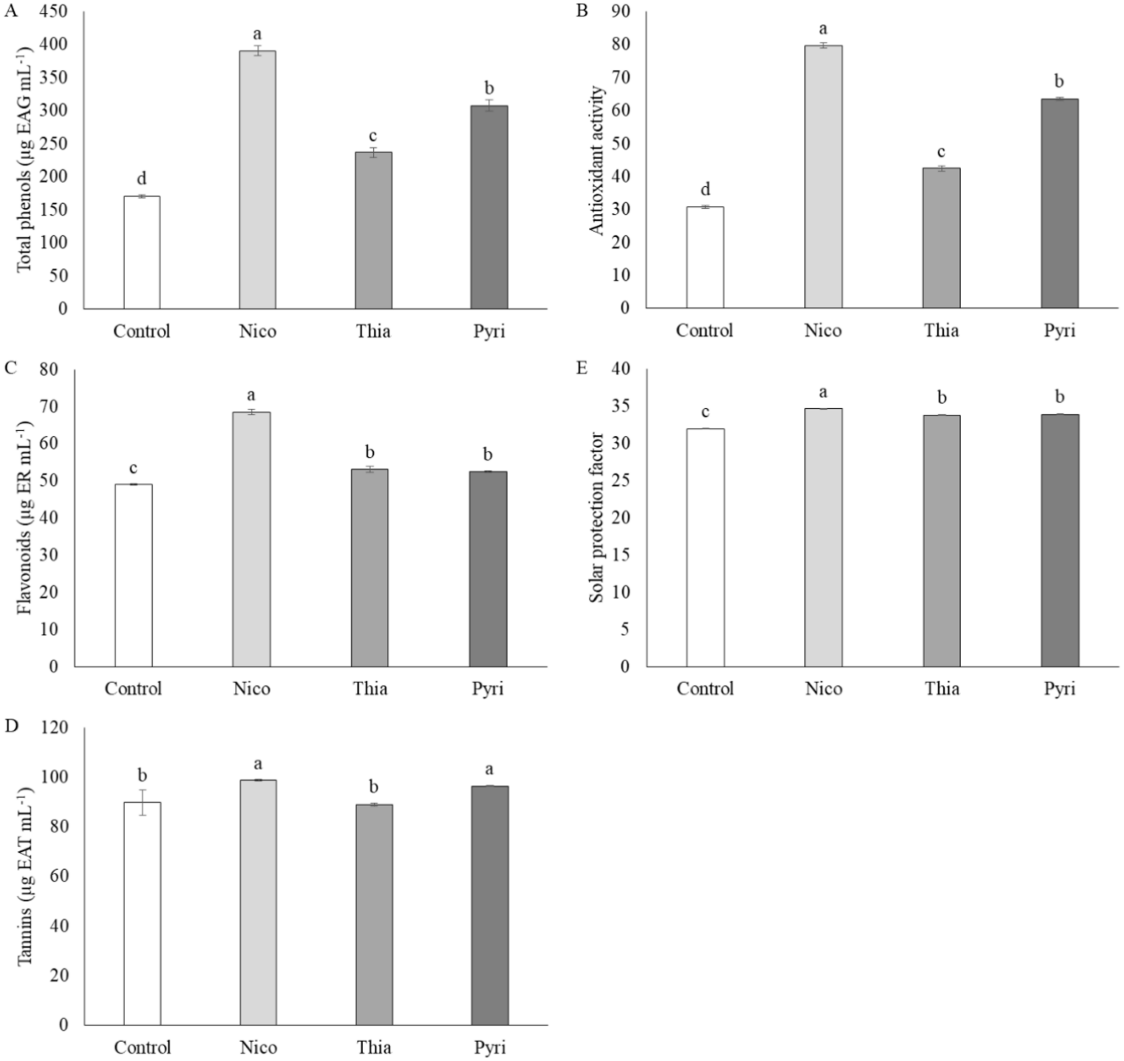
Total phenols **(A)**, flavonoids **(B)**, tannins **(C)**, antioxidant activity **(D)**, and solar protection factor of babyleaf basil grown under different vitamin treatments. Different letters on the bars indicate a significant difference between the means by the Tukey test (*p* < 0.05). Bars represent the mean values (*n* = 3 ± SD).

Regarding phenolic compound content, a positive effect of vitamin application was observed, with nicotinamide outperforming the other treatments, followed by pyridoxine and thiamine, both also superior to the control treatment (Figure 6A). For this variable, increases of 129.1, 80.8, and 39.1% were obtained when nicotinamide, pyridoxine, and thiamine were applied, respectively. A similar effect was obtained for antioxidant potential, flavonoid content, and sun protection factor (Figures 6B–D), with nicotinamide, pyridoxine, and thiamine resulting in gains of 159.2, 106.5, and 37.9% in antioxidant potential, 39.9, 7.1, and 8.4% in flavonoid content, and 8.5, 6.2, and 5.8% in sun protection factor, respectively. In addition, tannin levels were enhanced by nicotinamide and pyridoxine, with increases of 10 and 7.4%, respectively, compared to the control treatment.

**Figure 7:**
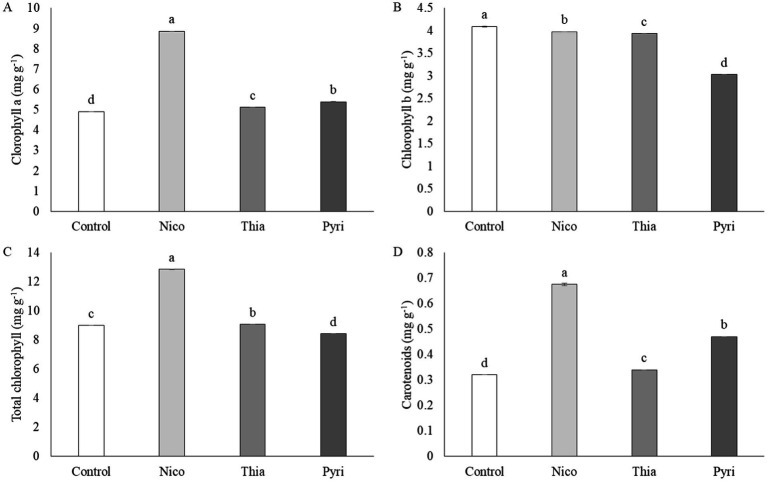
Chlorophyll a **(A)**, chlorophyll b **(B)**, total chlorophyll **(C)**, and carotenoids **(D)** of babyleaf basil grown under different vitamin treatments. Different letters on the bars indicate a significant difference between the means by the Tukey test (*p* < 0.05). Bars represent the mean values (*n* = 3 ± SD).

Leaf pigments were also altered by the treatments, with nicotinamide surpassing the other treatments for chlorophyll a (Figure 7A), increasing this variable by 80.6%. In contrast, for chlorophyll b, the control treatment showed superiority over the other treatments, with pyridoxine resulting in the lowest levels (Figure 7B). Nicotinamide also increased the total chlorophyll content of the plants and the carotenoid content, followed by pyridoxine in the latter. Compared to the control treatment, nicotinamide promoted gains of 110.7 and 46.9% in total chlorophyll and carotenoid content, respectively.

**Figure 8:**
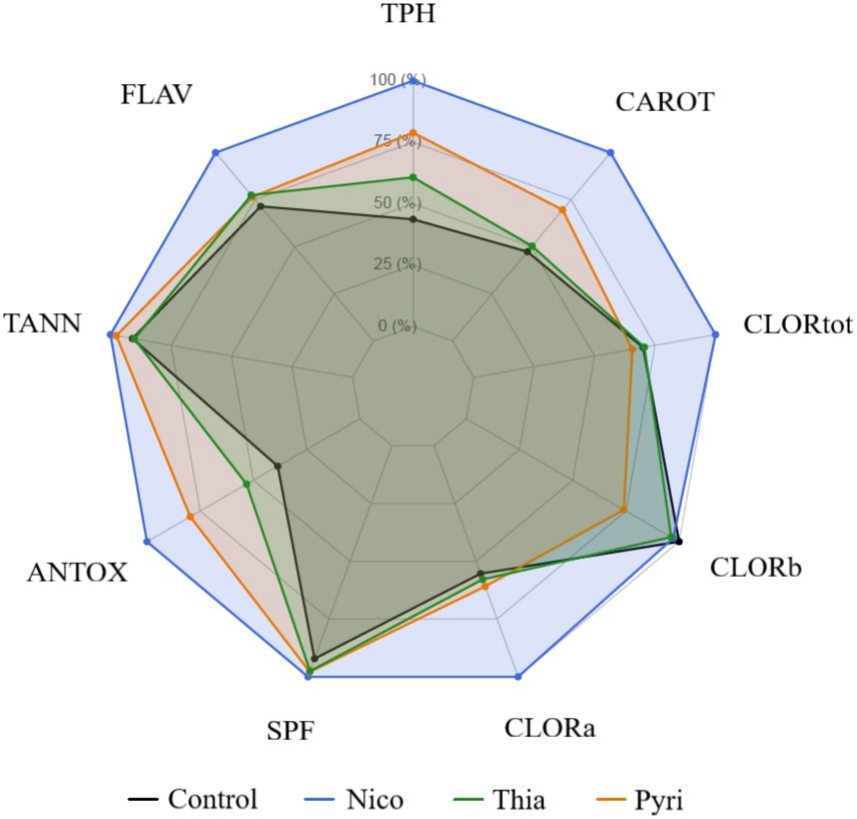
Radar chart of variables—total phenols (TPH), flavonoids (FLAV), tannins (TAN), antioxidant activity (ANT), sun protection factor (SPF), chlorophyll a (CLORa), chlorophyll b (CLORb), total chlorophyll (CLORtot), and carotenoids (CAROT) of baby leaf basil grown under different vitamin treatments.

The radar chart clearly shows the superiority of the nicotinamide treatment over the others, followed by the pyridoxine and thiamine treatments, respectively. It is noted that, except for chlorophyll b levels, the nicotinamide treatment outperformed the other treatments in all variables related to the accumulation of bioactive compounds and leaf pigments (Figure 8).

**Figure 9:**
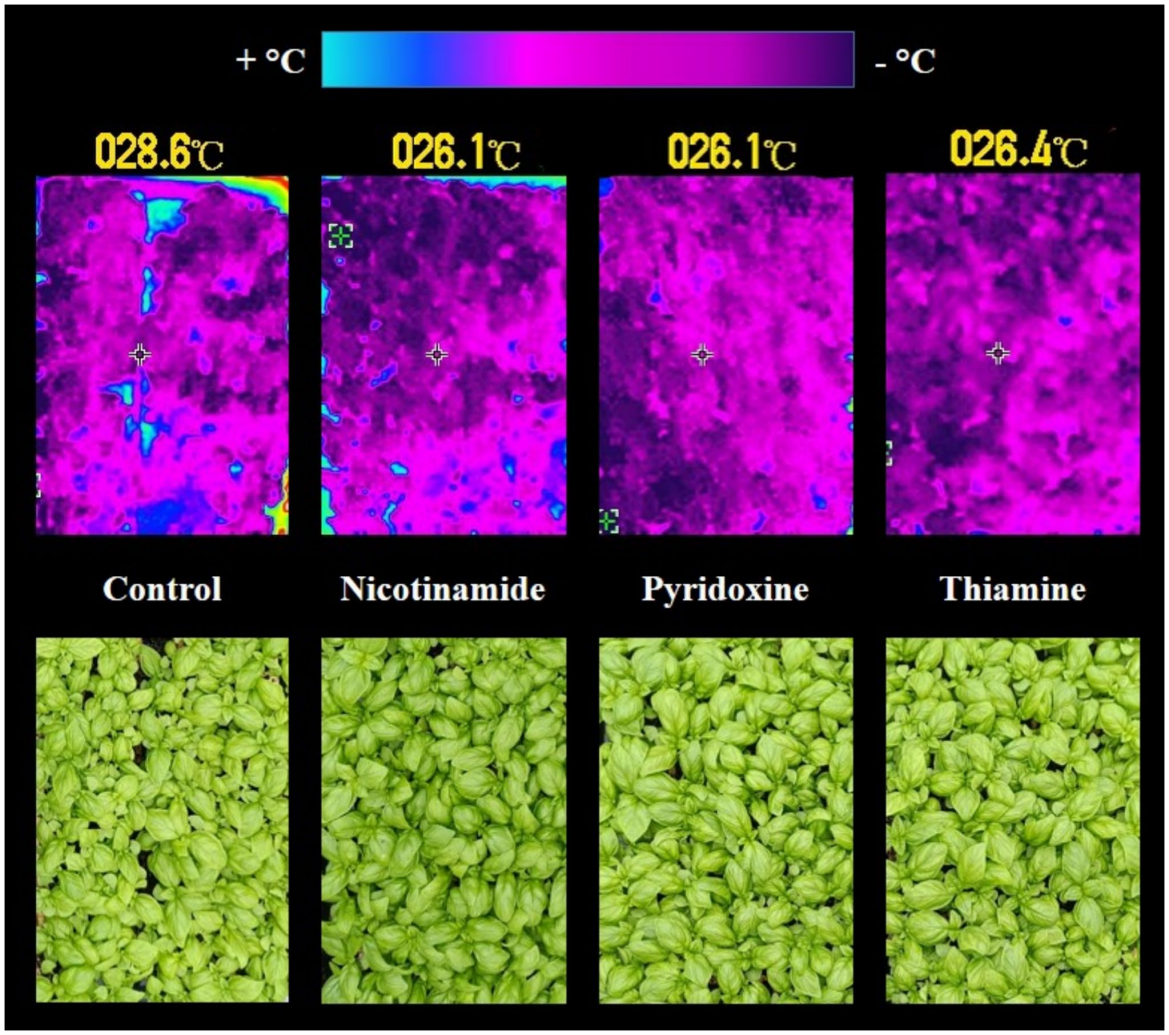
Thermographic image and comparative image of the leaf surface of babyleaf basil grown under different vitamin treatments.

The increase in leaf pigments provided by nicotinamide could be visually observed through the intensification of plant coloration. Also, the thermographic image identified that the treatments using vitamins reduced the leaf surface temperature by an average of approximately 2.4 °C (Figure 9).

**Figure 10:**
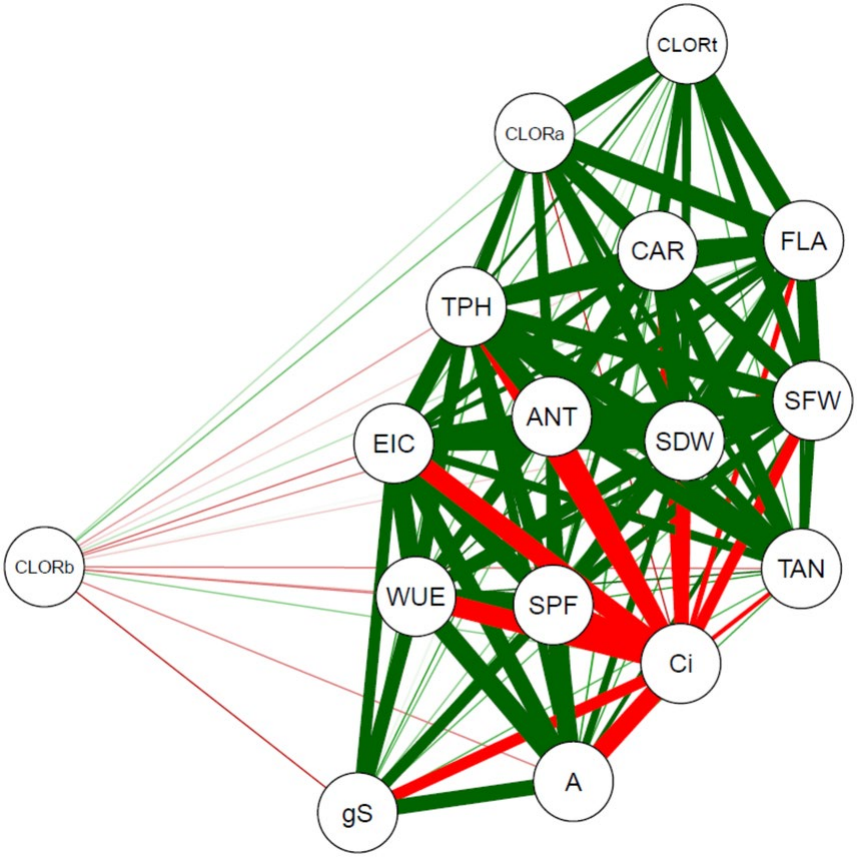
Pearson correlation network among variables—shoot fresh weight (SFW), shoot dry weight (SDW), intercellular CO_2_ content (Ci), stomatal conductance (gS), CO_2_ assimilation rate (A), water use efficiency (WUE), carboxylation efficiency (EIC), total phenols (TPH), flavonoids (FLAV), tannins (TAN), antioxidant activity (ANT), sun protection factor (SPF), chlorophyll a (CLORa), chlorophyll b (CLORb), total chlorophyll (CLORtot), and carotenoids (CAROT) of baby leaf basil grown under different vitamin treatments. Positive correlations were highlighted in green, and the line thickness was determined by a cut-off value of 0.7, corresponding to 70% reliability.

Strong correlations were found between most of the variables studied, including growth characteristics, production of bioactive compounds, leaf pigments, and gas exchange variables, of which only the intercellular CO_2_ content was negatively correlated with the vast majority of the aforementioned characteristics, while chlorophyll b showed only weak correlations (Figure 10).

**Figure 11:**
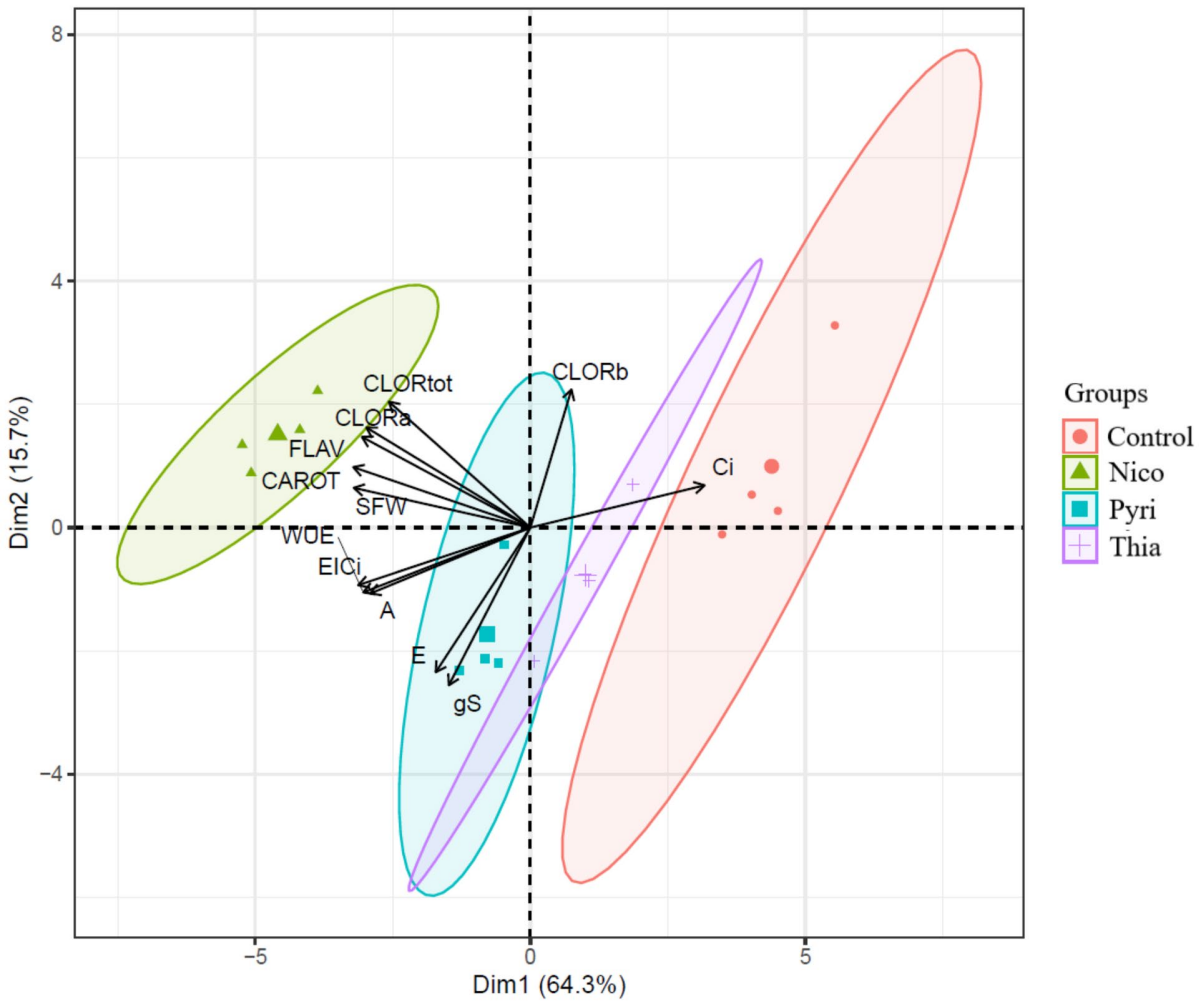
Principal component analysis of variables—shoot fresh weight (SFW), intercellular CO_2_ content (Ci), transpiration rate (E), stomatal conductance (gS), CO_2_ assimilation rate (A), water use efficiency (WUE), carboxylation efficiency (EICi), flavonoids (FLAV), chlorophyll a (CLORa), chlorophyll b (CLORb), total chlorophyll (CLORtot), and carotenoids (CAROT) of baby leaf basil grown under different vitamin treatments.

Principal component analysis revealed the formation of distinct groups among the treatments, with the nicotinamide treatment and the control group positioned in opposite quadrants. The strong influence of nicotinamide application on shoot fresh weight and the production of bioactive compounds and pigments (flavonoids, chlorophyll a, b and total, and carotenoids) was also evident, as was the influence of all vitamin treatments on gas exchange characteristics of transpiration rate (E), stomatal conductance (gS), CO_2_ assimilation rate (A), water use efficiency (WUE), carboxylation efficiency (EICi), but not for intercellular CO_2_ content, which showed a trend opposite to most other characteristics, leaning toward the control treatment (Figure 11).

## 4 Discussion

The observed effects of vitamin application on gas exchange are related to increased physiological efficiency in plants, as there is a reduction in CO_2_ content in the leaf mesophyll and a significant increase in assimilation rate, water use efficiency, and carboxylation efficiency (Figure 2).

As a result of the observed increase in gas exchange, there was greater development of vegetative organs, especially for plants cultivated under the influence of nicotinamide (Figure 3). This vitamin also provided the greatest accumulation of mass, both in the shoot and in roots (Figure 4), providing visually striking differences in the development of these organs (Figure 5).

For cultivation environments in tropical climates, or where cooling is not possible, increasing the capacity for gas exchange becomes a factor of great importance, aiming at maintaining leaf temperature at levels that allow the maintenance of physiological activities, through cooling by stomatal conductance and transpiration (17). This effect was also observed in the present study, since the plants treated with the different vitamins reduced the leaf surface temperature (Figure 7).

Regardless of the metabolites evaluated, nicotinamide also stood out, followed by pyridoxine and thiamine, respectively (Figure 6). In this sense, it is observed that the production of bioactive compounds by plants is related to the CO_2_ exchange rate, as well as the plant’s ability to use atmospheric CO_2_ as a substrate (14, 18). In the present study, it was found that the vitamins increased carboxylation efficiency (Figure 2E), while the CO_2_ concentration in the leaf mesophyll decreased (Figure 2A), indicating that the treatments were effective in improving the plant’s ability to use CO_2_ for the construction of carbon chains.

The combined effect of increased production capacity (Figure 4) and metabolite production (Figures 6, 8) establishes a significant positive correlation (Figures 10, 11), considering the demand from industrial sectors such as food and pharmaceuticals, which utilize the phytochemical properties of plants to manufacture various products (2, 3). In this sense, compounds with antioxidant characteristics are sought due to their high applicability to industrial processes, as well as their benefits to human health.

Phenolic compounds, flavonoids, and tannins have diverse applications, as they possess anti-inflammatory, immunoregulatory, neuroprotective, antiviral, and anticancer properties, among many others, including a strong antioxidant aspect. Therefore, their application in pharmaceutical and food products as additives is desirable (19, 20, 34). Thus, the results of our research highlighted the significant increase in the antioxidant potential (Figure 6B) and sun protection factor (Figure 6D) of extracts obtained from plants cultivated under the influence of vitamins (Figure 8).

It has been observed that the use of plant extracts has been increasingly sought for the production of cosmetics aimed at protecting against the deleterious effects of solar radiation (21). In food, extracts containing high levels of bioactive compounds and with relevant nutritional characteristics can be used beneficially for a wide variety of purposes, from maintaining the stability of a mixture to providing flavor and aroma in beverages and processed foods, and can also benefit the consumer through the consumption of products with high nutraceutical potential (22).

The combined effect of increased gas exchange capacity, coupled with the production of antioxidant compounds, also plays an important role in protecting the photosynthetic system of basil plants. The significant effect of nicotinamide on the levels of total chlorophyll a and carotenoids (Figures 7, 8), which were clearly distinguished by the visual difference in the green coloration of the leaves (Figure 9), also reflects the greater capacity to maintain pigments during their development. However, the reductions observed in chlorophyll b content following vitamin application (Figure 7B) may be attributed to the reorganization of light-harvesting complexes, as these vitamins are closely associated with enhanced photoprotection and improved photosynthetic performance (23, 24). Such adjustments in the photosynthetic apparatus often lead to an increase in the chlorophyll a/chlorophyll b ratio, reflecting a more efficient utilization of absorbed light energy rather than impairment of the photosynthetic machinery (25).

It has been observed that there is a positive correlation between the production of bioactive compounds, leaf pigments, gas exchange, and vegetative growth when plants are exposed to elicitors such as H_2_O_2_, implying a self-defense response against possible environmental stresses (7). However, the present study demonstrates that even in environments suitable for cultivation (Figure 1), this exposure is beneficial, increasing the physiological activity of the plants and stimulating their growth (Figures 10, 11).

The presence of high levels of leaf pigments also influences the nutraceutical quality of extracts, considering that the removal of these pigments can result in negative alterations of the antioxidant and anticancer properties of some species (26). In this sense, it is noteworthy that the inclusion of extracts with high pigment levels can help in various diseases, including some resulting from current habits, such as sedentary lifestyles (27).

Based on the findings presented above, the results of this study highlight the broad potential of vitamin applications in food production systems and in the generation of raw materials for diverse industrial sectors. The observed improvements in plant growth and quality contribute not only to the production of high-quality food but also to the supply of agricultural inputs with enhanced industrial value and processing efficiency. Nevertheless, given the relatively recent development of this research field, further studies are essential to investigate the limits and consistency of plant responses to vitamin applications across different production systems, environmental conditions, and crop species. Expanding this knowledge will strengthen the feasibility of large-scale implementation while providing a more robust scientific basis for recommending these compounds to small and medium-scale farmers. Ultimately, such advances may enhance production efficiency, increase the reliability of management practices, and contribute to greater economic security and sustainability within agricultural systems.

## 5 Conclusion

The use of B vitamins, especially nicotinamide, in hydroponic cultivation not only increases the growth and productivity of young basil plants but can also be used as an elicitor to stimulate the production of metabolites of food and industrial interest.

This technology assists in cultivation in controlled environments, where the goal is to produce high-quality nutraceutical crops, helping to supply products rich in bioactive compounds that promote health and can be used in the food, cosmetic, and pharmaceutical industries.

Gas exchange characteristics, levels of bioactive compounds, antioxidant activity, and leaf pigment production are strongly and positively correlated and directly affect the yield of young basil plants.

## Data Availability

The original contributions presented in the study are included in the article/Supplementary material, further inquiries can be directed to the corresponding authors.
